# Supplementary material for: Voltage-Seq: all-optical postsynaptic connectome-guided single-cell transcriptomics
Source: Nat Methods. 2023 Jul 20;20(9):1409–16. doi: 10.1038/s41592-023-01965-1 (PMC10482676; doi:10.1038/s41592-023-01965-1)
Supplement: Supplementary file 1 — Reporting Summary [file 41592_2023_1965_MOESM1_ESM.pdf]

nature portfolio

Corresponding author(s): Janos Fuzik Ph.D. (janos.fuzik@ki.se)

Last updated by author(s): Jun 13, 2023

Reporting Summary

Nature Portfolio wishes to improve the reproducibility of the work that we publish. This form provides structure for consistency and transparency in reporting. For further information on Nature Portfolio policies, see our Editorial Policies and the Editorial Policy Checklist.

Statistics

For all statistical analyses, confirm that the following items are present in the figure legend, table legend, main text, or Methods section.

n/a	Confirmed	
☐	☒	The exact sample size (*n*) for each experimental group/condition, given as a discrete number and unit of measurement
☐	☒	A statement on whether measurements were taken from distinct samples or whether the same sample was measured repeatedly
☐	☒	The statistical test(s) used AND whether they are one- or two-sided<br>*Only common tests should be described solely by name; describe more complex techniques in the Methods section.*
☒	☐	A description of all covariates tested
☒	☐	A description of any assumptions or corrections, such as tests of normality and adjustment for multiple comparisons
☐	☒	A full description of the statistical parameters including central tendency (e.g. means) or other basic estimates (e.g. regression coefficient) AND variation (e.g. standard deviation) or associated estimates of uncertainty (e.g. confidence intervals)
☐	☒	For null hypothesis testing, the test statistic (e.g. *F*, *t*, *r*) with confidence intervals, effect sizes, degrees of freedom and *P* value noted<br>*Give P values as exact values whenever suitable.*
☒	☐	For Bayesian analysis, information on the choice of priors and Markov chain Monte Carlo settings
☐	☒	For hierarchical and complex designs, identification of the appropriate level for tests and full reporting of outcomes
☒	☐	Estimates of effect sizes (e.g. Cohen's *d*, Pearson's *r*), indicating how they were calculated

Our web collection on statistics for biologists contains articles on many of the points above.

Software and code

Policy information about availability of computer code

Data collection	HCImage Live 4.6.0, pClamp 10.4, Matlab 2021b
Data analysis	Matlab 2021b; VoltView 1.0 (https://zenodo.org/record/8030176)

For manuscripts utilizing custom algorithms or software that are central to the research but not yet described in published literature, software must be made available to editors and reviewers. We strongly encourage code deposition in a community repository (e.g. GitHub). See the Nature Portfolio guidelines for submitting code & software for further information.

Data

Policy information about availability of data

All manuscripts must include a data availability statement. This statement should provide the following information, where applicable:

- Accession codes, unique identifiers, or web links for publicly available datasets
- A description of any restrictions on data availability
- For clinical datasets or third party data, please ensure that the statement adheres to our policy

We gave access to "minimum dataset" to test our VoltView 1.0 analysis, we made example raw all-optical voltage imaging data available at https://zenodo.org/record/8030176 , and we included source data for Figure 2 and Figure 3 with the manuscript. We deposit RNA-transcriptomic data of Voltage-Seq experiments to https://www.ebi.ac.uk/fg/annotare/help/submit_exp.html

Human research participants

Policy information about studies involving human research participants and Sex and Gender in Research.

Reporting on sex and gender	N/A
Population characteristics	N/A
Recruitment	N/A
Ethics oversight	N/A

Note that full information on the approval of the study protocol must also be provided in the manuscript.

Field-specific reporting

Please select the one below that is the best fit for your research. If you are not sure, read the appropriate sections before making your selection.

☒ Life sciences ☐ Behavioural & social sciences ☐ Ecological, evolutionary & environmental sciences

For a reference copy of the document with all sections, see nature.com/documents/nr-reporting-summary-flat.pdf

Life sciences study design

All studies must disclose on these points even when the disclosure is negative.

Sample size	For our largest data body we used 7 mice, and voltage imaged ~7000 neurons in them. We continued the experiments until we had sufficient spatial coverage of the voltage-imaged area (at least 40 cells/200umx200umx200um tissue volumes).
Data exclusions	No data were excluded.
Replication	Multiple animals were voltage-imaged at the same XYZ coordinates in the brain and results were cross-compared to validate the lack of batch-effect. Experiments were done in the period of ~4 months, and were replicated every 1-2 weeks successfully depending on sufficient expression of the voltage sensor in the neurons to image.
Randomization	We conducted mainly proof of principal experiments without multiple groups, thus comparisons and randomization were not crucial
Blinding	Blinding was not relevant during data collection because we conducted experiments in the same manner, tile-imaged the same coordinates to cover the same brain areas. During analysis, the same analysis script was used to all the data, blinding was not relevant either.

Reporting for specific materials, systems and methods

We require information from authors about some types of materials, experimental systems and methods used in many studies. Here, indicate whether each material, system or method listed is relevant to your study. If you are not sure if a list item applies to your research, read the appropriate section before selecting a response.

Materials & experimental systems

n/a	Involved in the study	
☐	☒	Antibodies
☒	☐	Eukaryotic cell lines
☒	☐	Palaeontology and archaeology
☐	☒	Animals and other organisms
☒	☐	Clinical data
☒	☐	Dual use research of concern

Methods

n/a	Involved in the study	
☒	☐	ChIP-seq
☒	☐	Flow cytometry
☒	☐	MRI-based neuroimaging

Antibodies

Antibodies used	NeuN (Millipore, MAB377, clone A60, 1:1000 dil); Alexa Fluor 633-conjugated streptavidin (Thermo Fisher, S21375, 1:1000 dil); Cy™5 AffiniPure Donkey Anti-Mouse IgG (H+L) (Jackson, Code:715-175-151, 1:500 dil)
Validation	Anti-NeuN Antibody, clone A60 detects level of NeuN and has been published and validated for use in FC, IC, IF, IH, IH(P), IP and WB

Validation	(https://www.merckmillipore.com/SE/en/product/Anti-NeuN-Antibody-clone-A60,MM_NF-MAB377).

Animals and other research organisms

Policy information about studies involving animals; ARRIVE guidelines recommended for reporting animal research, and Sex and Gender in Research

Laboratory animals	All mice were 3-5 month old; wild-type: C57BL/6J (Charles River Laboratories), VGAT-Cre: B6J.129S6(FVB)-Slc32a1tm2(cre)Lowl/MwarJ, Jackson stock no. 028862.
Wild animals	No wild animals were used to produce the data of the manuscript.
Reporting on sex	We used males to minimize potential data variance rising from the sex-differences, because we used fewer animals than for a behavioral or physiological study to further emphasize the power of throughput of our methodology.
Field-collected samples	No field-collected samples were used in the study.
Ethics oversight	All procedures were approved and performed in accordance and compliance with the guidelines of the Stockholm Municipal Committee (approval no. N166/15 and 7362-2019).

Note that full information on the approval of the study protocol must also be provided in the manuscript.
